# Inhibitory Effect of Chemical Constituents Isolated from* Artemisia iwayomogi* on Polyol Pathway and Simultaneous Quantification of Major Bioactive Compounds

**DOI:** 10.1155/2017/7375615

**Published:** 2017-04-23

**Authors:** Yoon Kyoung Lee, Eun Young Hong, Wan Kyunn Whang

**Affiliations:** Pharmaceutical Botany Laboratory, College of Pharmacy, Chung-Ang University, Heukseok-dong, Dongjak-gu, Seoul 156-756, Republic of Korea

## Abstract

Blocking the polyol pathway plays an important role preventing diabetic complications. Therefore, aldose reductase (AR) and advanced glycation endproducts (AGEs) formation has significant effect on diabetic complications.* Artemisia iwayomogi* has long been used as treatment of various diseases in Korea. However, no literatures have reported on AR and AGEs formation inhibitory activities of* A. iwayomogi*. For these reasons, we aimed to assess that* A. iwayomogi* had potential as anti-diabetic complications agents. We led to isolation of two coumarins (**1** and** 2**), nine flavonoids (**3**–**11**), five caffeoylquinic acids (**12**–**16**), three diterpene glycosides (**17**–**19**), and one phenolic compound (**20**) from* A. iwayomogi*. Among them, hispidulin (**4**), 6-methoxytricin (**6**), arteanoflavone (**7**), quercetin-3-gentiobioside (**10**), 1,3-di-*O*-caffeoylquinic acid (**13**), and suavioside A (**18**) were first reported on the isolation from* A. iwayomogi*. Not only two coumarins (**1** and** 2**), nine flavonoids (**3**–**11**), and five caffeoylquinic acids (**12**–**16**) but also extracts showed significant inhibitor on AR and AGEs formation activities. We analyzed contents of major bioactive compounds in Korea's various regions of* A. iwayomogi*. Overall, we selected Yangyang, Gangwon-do, from June, which contained the highest amounts of bioactive compounds, as suitable areas for cultivating* A. iwayomogi* as preventive or therapeutic agent in the treatment of diabetic complications.

## 1. Introduction

Aldose reductase (AR) belongs to the aldo-keto reductase family. It is the first and rate-controlling enzyme in the polyol pathway that reduces glucose to sorbitol using nicotinamide adenine dinucleotide 2′-phosphate (NADPH) as a cofactor [1, 2]. Although blood sugar is high, the polyol pathway converts the excess glucose into sorbitol and then fructose. This accumulation of sorbitol and fructose has been demonstrated to be responsible for diabetic complications, including nephropathy, cataracts, neuropathy, and retinopathy [3, 4]. In particular, sorbitol is implicated in the pathogenesis of sugar cataracts, while the accumulation of fructose induces the formation of advanced glycation endproducts (AGEs), which are strongly implicated in diabetic complications and Alzheimer's disease [5]. Therefore, research on AR and AGEs formation inhibition is on the rise. There is a wide range of literature demonstrating that the developments of AR and AGEs formation are blocked by natural sources, especially plants that have an enormous content of bioactive compounds [6, 7].

*Artemisia iwayomogi*, locally called haninjin or dowijigi, is a member of the Compositae family and a perennial herb mainly found in Korea.* A. iwayomogi* has long been used in Korea in vegetables and foods such as tea, rice cake, and soup and also used for the treatment of various diseases including hepatitis, inflammation, and immune-related diseases as a protection for the liver, and a diuretic [8–10]. Previous studies have reported the isolation of scopoletin, esculetin 6-methylether, scopolin, *β*-sitosterol, chlorogenic acid, quebrachitol, essential oils, fatty acids, sesquiterpene lactones, and flavonoids in* A. iwayomogi *[11–14]. Among them, scopolin modulated the expression of obesity-associated genes and was shown to have pharmacological effects on obesity, fatty liver, and diabetes [15].* A. iwayomogi* has also demonstrated various biological activities. For example, methanol extracts of* A. iwayomogi* were shown to act as a scavenger of peroxynitrile, a compound involved in inducing or maintaining many diseases, including inflammation and aging [16]. Methanol extract of* A. iwayomogi* also inhibits nitric oxide in the production of lipopolysaccharide activated macrophages [17]. In other studies, a water-soluble carbohydrate fraction from* A. iwayomogi* suppressed spontaneous or 2,3,7,8-tetrachlorodibenzo-*p*-dioxin-induced apoptotic death of mouse thymocytes by downregulating Fas gene expression [18, 19]. A water-soluble carbohydrate fraction from* A. iwayomogi* also repressed pulmonary eosinophilia and Th2-type cytokine production in ovalbumin-induced allergic asthma via downregulation of TNF-*α* expression in the lungs [20], showed antitumor, immunomodulating activities [9], and modulated the functional differentiation of bone marrow-derived dendritic cells [21].

However,* A. iwayomogi*'s ability to inhibit AR and AGEs formation has never been previously studied. We investigated various components found in* A. iwayomogi* and their inhibitory activities on AR and AGEs formation. Our study also determined the bioactive compounds present in* A. iwayomogi *by region in Korea using an HPLC-PAD. Based on our study, we selected suitable areas for cultivating* A. iwayomogi *as medicinal foods.

## 2. Materials and Methods

### 2.1. Plant Materials

The aerial parts of* A. iwayomogi* were purchased from Kyung-Dong market, Seoul, Korea. Moreover, the aerial parts of* A. iwayomogi *were collected from Seoul, Gyeongii-do, Chungcheongbuk-do, Jeollabuk-do, Jeollanam-do, Gyeongsangbuk-do, Gyeongsangnam-do, Gangwon-do, and Jeju-do Korea for analysis. Professor Whang Wan Kyunn identified* A. iwayomogi* which was bought in Kyung-Dong market and collected in various specimens.

### 2.2. Instruments and Reagents

Toluene, ethyl formate, chloroform (CHCl_3_), ethyl acetate (EtOAc), methanol (MeOH), ethanol (EtOH), distilled water, and formic acid were used as the open column chromatography and thin layer chromatography. Open column chromatography was used with Amberlite gel (Nippon Rensui Co., Japan), silica gel (200~400 mesh; Merck Co., Germany), Sephadex LH-20 (25–100 *μ*m; Pharmacia, Sweden), MCI CHP 20P (Supelco, USA), and ODS gel (400–500 mesh; Waters, USA). Thin layer chromatography was conducted with a Kiesel gel 60 F_254_ (Merck Co., Darmstadt, Germany) and plates (silica gel, 0.25 mm layer on aluminum sheets), and compounds were profiled by spraying with 10% H_2_SO_4_ in EtOH, followed by heating to 110°C. Dimethyl sulfoxide-*d*_6_ (DMSO-*d*_6_), methanol-*d*_6_ (CD_3_OD), and pyridine-*d*_6_ were used as the NMR solution. MS was performed using fast atom bombardment mass spectrometry (FAB-MS) conducted using a JEOL JMS-600W (Tokyo, Japan) mass spectrometer, a Q-TOF Synapt G2 apparatus (Waters, Milford, Massachusetts, USA) equipped with electrospray ionization (ESI), and electrospray ionization hybrid linear trap-quadruple-orbitrap (ESI/LTQ-Orbitrap) using an Ultimate 3000 RSLC (Thermo, Germany). ^1^H and ^13^C-nuclear magnetic resonance (NMR) spectra were conducted at 600 MHz and 150 MHz, respectively, with a Varian Gemini 2000 (USA) spectrometer using tetramethylsilane (TMS) as an internal standard. Chemical shifts are expressed as parts per million (ppm) on the *δ* scale, and coupling constants (*J*) are showed in Hertz. HPLC was conducted using Empower Pro 2.0 software and determination was performed using a Waters 2695 system pump with a Waters 996 Photodiode array detector (USA). The separation column was a Waters Sunfire™ column C18 (4.6 × 250 mm, 5 *μ*m). HPLC-grade solvents such as acetonitrile (ACN), methanol (MeOH), and distilled water (H_2_O) were purchased from J. T. Baker® (Phillipsburg, PA, USA). HPLC-grade formic acid and dimethyl sulfoxide (DMSO) were obtained from DEAJUNG Chemical (Siheung, Gyeonggi, Korea). Reagents and solvents including dl-glyceraldehyde, potassium phosphate buffer, nicotinamide adenine dinucleotide 2′-phosphate (*β*-NADPH), sodium phosphate buffer, sodium azide, 3,3-tetramethyleneglutaric acid (TMG), bovine serum albumin, aminoguaidine (AG), glucose, and fructose were purchased from Sigma-Aldrich (St. Louis, MO, USA).

### 2.3. Extraction, Fractionation, and Isolation from* A. iwayomogi*

The dried and powdered aerial parts of* A. iwayomogi *(5 kg) were extracted with 50% EtOH (20 L × 3) at room temperature. The filtrate was concentrated to dryness (692.1 g) in vacuo, suspended in water (H_2_O), and then partitioned using CHCl_3_ (97.6 g). After partitioning, the water fraction (456.7 g) was chromatographed on a Amberlite XAD-2 using water, 30% methanol, 60% methanol, and 100% methanol. The result yielded water (240 g), 30% methanol (92.18 g), 60% methanol (49.31 g), and 100% methanol (70.24 g) fractions.

The CHCl_3_ fraction (97.6 g) was chromatographed with a silica gel solvent system to increase the elution power of CHCl_3_ : MeOH (20 : 1 to 0 : 100) and yielded ten subfractions. Subfraction 2 (25.62 g) was separated using Sephadex LH-20 column chromatography with 50% MeOH to obtain fractions 2-1~2-11. Compound** 1 **(1.16 g) was isolated from fraction 2-2. In addition, separation of subfraction 2-3 (6.39 g) using an MCI gel with 50% MeOH solvent system yielded fourteen fractions. Subfraction 2-3-10 led to isolation of compound** 3** (34.4 mg). Fraction 2-3-6 (632.5 mg) was separated using ODS column chromatography with a 60% MeOH elution solvent system to collect fractions 2-3-6-1~2-3-6-4. Compound** 5** (97.5 mg) was isolated from subfraction 2-3-6-2 by recrystallization, and then a portion of subfraction 2-3-6-2 (324.2 mg) was used on a Sephadex LH-20 (50% MeOH), to yield compound** 6 **(13.2 mg). Subfraction 2-3-9 (448.4 mg) was separated using ODS (60% MeOH), MCI (50% MeOH), and Sephadex LH-20 (40% EtOH), which yielded compound** 7 **(17.5 mg). Fraction 3 (2.38 g) was applied to a Sephadex LH-20 column chromatography with a gradient solvent system of 50% to 100% MeOH and yielded fourteen fractions. Fraction 3-9 (96.8 mg) was separated using ODS column chromatography with 70% MeOH, which obtained six fractions (3-9-1~3-9-6). Subfraction 3-9-3 (20 mg) was chromatographed on a Sephadex LH-20 using 50% MeOH to yield compound** 4 **(19.9 mg).

A portion of the 100% MeOH fraction (15 g) was separated using a Sephadex LH-20 column chromatography with a gradient elution solvent system of 20% to 100% MeOH to give eighteen subfractions. Subfraction 3 (0.93 g) was applied to ODS column chromatography using a 10% MeOH solvent system to yield compound** 12 **(37.9 mg). Subfraction 16 (0.99 g) was separated using a ODS column chromatography with 20% MeOH, and ten fractions (16-1~16-10) were collected. Fraction 16-6 (134.3 mg) led to the isolation of compound** 14** (70.0 mg) by recrystallization. Fraction 16-5 (395.7 mg) was separated by a Sephadex LH-20 using an eluent of 40% EtOH, which yielded compounds** 15** (272.2 mg) and** 16** (72.2 mg).

The 60% MeOH fraction (49.31 g) was separated using a Sephadex LH-20 column chromatography with a gradient of 40% MeOH to 100% MeOH, which yielded twelve subfractions. Subfraction 1 (6.7 g) was separated by an MCI gel using a 50% MeOH to 100% MeOH solvent system, which yielded fifteen fractions (1-1~1-15). Fractions 1-8 (331.6 mg), 1-9 (101.6 mg), and 1-10 (157.5 mg) were isolated by recrystallization to yield compounds** 17 **(32.7 mg),** 18** (9.2 mg), and** 19** (28.6 mg), respectively. Subfraction 3 (923.4 mg) was separated using an ODS column chromatography, and seven fractions (3-1~3-7) were collected after elution with 40% MeOH. Fraction 3-3 (140.4 mg) was separated by MCI column chromatography using an eluent of 50% MeOH, leading to the isolation of compound** 20**. Subfraction 5 (1.48 g) was separated using an MCI gel and eluted with 50% MeOH, which yielded compound** 8** (281.7 mg). A portion of fraction 5-3 (166.2 mg) was applied to an MCI gel and eluted with 40% MeOH. Fraction 5-3-3 (106 mg) was separated by a Sephadex LH-20 column chromatography using an eluent of 50% EtOH to yield compounds** 10** (23.1 mg) and** 11** (36.8 mg). Subfraction 7 (806.2 mg) and subfraction 8 (3. 77 g) were chromatographed on an MCI gel using 40% MeOH, to give fractions 7-1~7-10 and 8-1~8-12, respectively. Compound** 9** (76.6 mg) was isolated from fraction 7-4 (108.8 mg) using a Sephadex LH-20 eluting with 50% EtOH. Subfraction 8-2 (3.77 g) was separated using a Sephadex LH-20 eluting with 40% MeOH. Fraction 8-2-3 was applied to a Sephadex LH-20 column chromatography using 20% EtOH, which yielded compound** 13 **(111.1 mg).

A portion of the 30% MeOH fraction (14 g) was chromatographed on a Sephadex LH-20 column chromatography using a solvent system of 20% MeOH to 100% MeOH, which yielded seven subfractions. Fraction 2 (7.12 mg) was separated using an ODS column chromatography, and fifteen fractions (2-1~2-15) were collected after elution with 5% MeOH. Subfraction 2-6 was applied to an MCI gel using 5% EtOH, which yielded compound** 2 **(171.4 mg).

### 2.4. Measurement of AR Inhibitory Activity

Rat lenses (one lens per 0.5 mL sodium buffer) were obtained from Sprague-Dawley rats (weighing 250~280 g). The rat lenses were homogenized in 0.1 M sodium phosphate buffer (pH 6.2) and centrifuged at 10,000 rpm (4°C, 20 min). After centrifugation, the supernatant was used as an enzyme. AR activity was spectrophotometrically determined by measuring the decrease in the absorption of *β*-NADPH at 340 nm for a 4 min period using dl-glyceraldehydes as substrates [22]. The assay mixture contained 1.6 mM *β*-NADPH, 0.1 M potassium phosphate buffer (pH 7.0), AR homogenate, 4 M ammonium sulfate, 0.025 M dl-glyceraldehyde, and the sample in 100% DMSO. The total volume of the assay mixture was 0.3 mL and the reaction was performed in a 96-well plate. 3,3′-Tetramethylene glutaric acid (TMG), a typical AR inhibitor, was used as a positive control.

### 2.5. Measurement of AGEs Formation Inhibitory Activity

Inhibition of AGEs formation was determined by an assay containing bovine serum albumin (10 mg/mL) in 50 mM phosphate buffer (pH 7.4) with 0.02% sodium azide, to which 0.4 M fructose and glucose was added. The assay mixture was incubated at 60°C for 2 days. After incubating, the reaction was measured on Fluorescence in a 96-black-well plate (excitation wavelength 350 nm, emission wavelength 450 nm) [23]. Aminoguaidine (AG) was used as a positive control.

### 2.6. For AR and AGEs Formation Inhibition Assays, the Activity Was Calculated

Inhibition (%): (Ac − As/Ac) × 100. In AR inhibition activity, Ac is absorbance of control and As is absorbance of samples. In AGEs formation inhibition activity, Ac is fluorescence of control and As is fluorescence of samples. IC_50_ is the concentration of inhibitor that gives a 50% inhibition in enzyme activity. IC_50_ values were calculated from the least-squares regression line of the log of concentration plotted against residual activity. All assays were performed in triplicate. Data was presented as mean ± standard deviation (SD).

### 2.7. Sample Preparation for HPLC

Samples of* A. iwayomogi *taken from the various regions in Korea and Yangyang, Gangwon-do, monthly between June and October were dissolved in 50% EtOH (20 mg/mL). The resulting solution was filtered with a 0.45 *μ*m syringe filter. The resulting solution was used for HPLC analysis.

### 2.8. HPLC Conditions

A Waters Sunfire™ column C18 (4.6 × 250 mm, 5 *μ*m) was used for the determination of compounds** 1**,** 2**,** 8**,** 10**,** 11**,** 12**,** 14**, and** 15**. The mobile phase consisted of 0.1% formic acid (solvent A) and ACN (solvent B). The gradient solvent system was solvents A and B (85 : 15) and increased in linear gradients to 84 : 16 for 5 min, to 72 : 28 for 5 min, then to 69 : 31 for 5 min, and finally to 40 : 60 for 5 min. The injection volume was 10 *μ*L and the flow rate was 0.9 mL/min. The UV spectra were recorded at 330 nm for quantification of compounds. All injections were performed in triplicate.

### 2.9. Calibration Curve

Stock solutions of compounds** 1**,** 2**,** 8**,** 10**,** 11**,** 12**,** 14**, and** 15** in 50% EtOH were prepared in several concentrations. Calibration curves of the eight standards were calculated using concentration (*X*, *μ*g/mL), peak area (*Y*), and mean value (*n* = 3) ± standard deviation. Contents of the analyte solutions were then determined from the calibration curves.

### 2.10. Determination of Limit of Detection (LOD) and Limit of Quantification (LOQ)

Quantification of the HPLC method for compounds** 1**,** 2**,** 8**,** 10**,** 11**,** 12**,** 14**, and** 15** as a standard compound was determined by LOD and LOQ. The LOD and LOQ were defined as detectable concentration of the compound with a signal to noise (*S*/*N*) ratio of ≥3.3 and ≥10, respectively. The percent recovery was evaluated by calculating the ratio of the amount detected versus the amount added. The quantity of analysis was subsequently obtained from the calibration curve.

## 3. Results

### 3.1. Identification of Compounds 1–20 Isolated from* A. iwayomogi*

**Figure 1:**
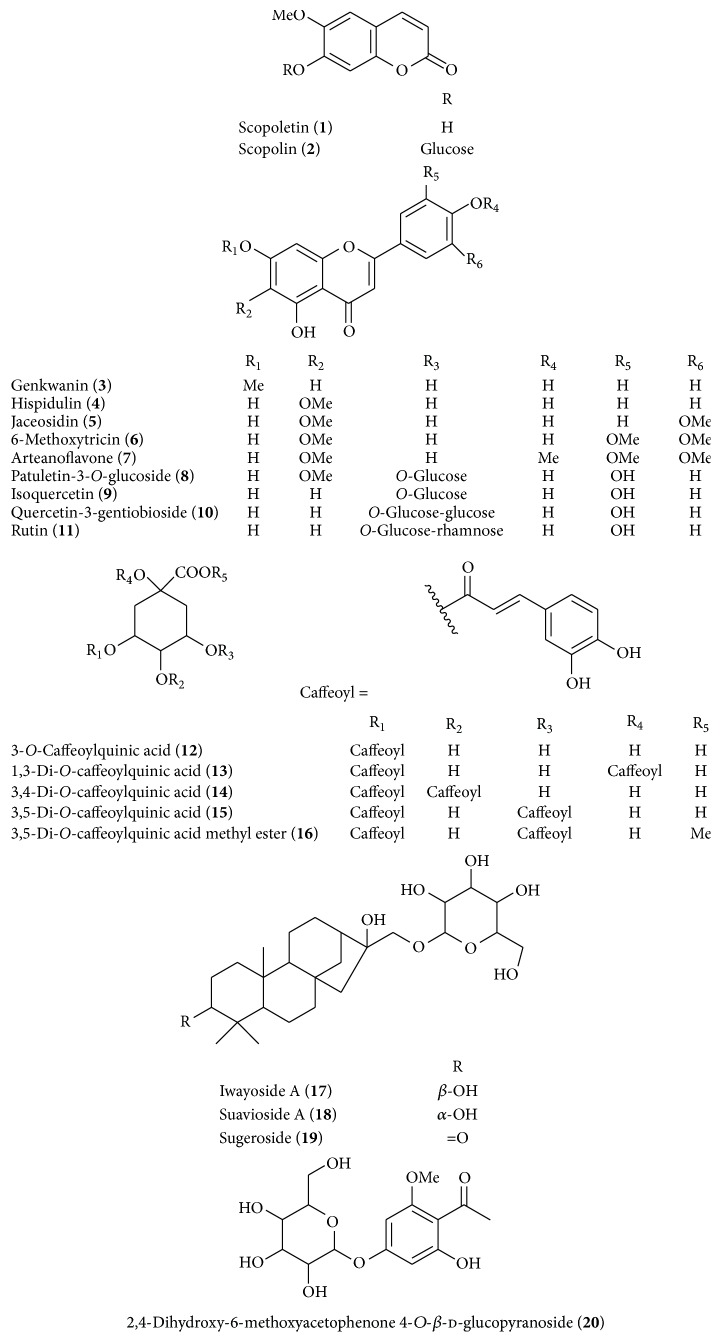
Structures of the compounds** 1–20**.

Chromatographic separation of the 50% EtOH extraction from* A. iwayomogi* led to the isolation of two coumarins (**1** and** 2**), nine flavonoids (**3**–**11**), five caffeoylquinic acids (**12**–**16**), three diterpene glycosides (**17**–**19**), and one phenolic compound (**20**) (Figure 1). Compounds** 1**–**20** isolated from* A. iwayomogi* were identified as scopoletin (**1**) [11, 24], scopolin (**2**) [11, 24], genkwanin (**3**) [11], hispidulin (**4**) [25], jaceosidin (**5**) [11, 26], 6-methoxytricin (**6**) [26], arteanoflavone (**7**) [27], patuletin-3-*O*-glucoside (**8**) [28], isoquercetin (**9**) [24, 29], quercetin-3-gentiobioside (**10**) [29], rutin (**11**) [30], 3-*O*-caffeoylquinic acid (**12**) [24], 1,3-di-*O*-caffeoylquinic acid (**13**) [31], 3,4-di-*O*-caffeoylquinic acid (**14**) [32], 3,5-di-*O*-caffeoylquinic acid (**15**) [24, 32], 3,5-di-*O*-caffeoylquinic acid methyl ester (**16**) [32], iwayoside A (**17**) [33], suavioside A (**18**) [34], sugeroside (**19**) [34], and 2,4-dihydroxy-6-methoxyacetophenone 4-*O*-*β*-d-glucopyranoside (**20**) [11] by comparison with the spectroscopic data (^1^H-NMR, ^13^C-NMR, and MS) from the literature.

Compound** 1**: C_10_H_8_O_4_; Q-TOF-MS* m/z*: 193.0490 [M+H]^+^; ^1^H-NMR (600 MHz, DMSO-*d*_6_) *δ*: 7.93 (1H, d,* J *= 9.6 Hz, H-4), 7.24 (1H, s, H-5), 6.81 (1H, s, H-8), 6.25 (1H, 3,* J *= 9.0 Hz, H-3), 3.85 (3H, s, 6-OMe); ^13^C-NMR (150 MHz, DMSO-*d*_6_) *δ*: 162.5 (C-2), 152.8 (C-7), 151.3 (C-9), 147.0 (C-6), 146.3 (C-4), 113.5 (C-3, 10), 112.4 (C-5), 104.5 (C-8), 57.8 (6-OMe).

Compound** 2**: C_16_H_18_O_9_; Q-TOF-MS* m/z*: 353.0869 [M−H]^−^; ^1^H-NMR (600 MHz, DMSO-*d*_6_): 8.00 (1H, d,* J *= 9.0 Hz, H-4), 7.33 (1H, d,* J *= 1.8 Hz, H-5), 7.19 (1H, s, H-8), 6.36 (1H, 3,* J *= 9.0 Hz, H-3), 5.12 (1H, d,* J *= 7.2 Hz, Glc H-1), 3.86 (3H, s, 6-OMe); ^13^C-NMR (150 MHz, DMSO-*d*_6_): 161.0 (C-2), 150.2 (C-7), 147.2 (C-9), 146.3 (C-6), 144.6 (C-4), 113.7 (C-3), 112.7 (C-10), 110.0 (C-5), 103.4 (C-8), 100.0 (Glc C-1), 77.4 (Glc C-3), 76.9 (Glc C-5), 73.3 (Glc C-2), 69.9 (Glc C-4), 60.9 (Glc C-6), 56.4 (6-OMe).

Compound** 3**: C_16_H_12_O_5_; ESI/LTQ-Orbitrap-MS* m/z*: 285.08 [M+H]^+^; ^1^H-NMR (600 MHz, DMSO-*d*_6_): 12.93 (5-OH), 7.96 (2H, d,* J *= 9.0 Hz, H-2′, 6′), 6.94 (2H, d,* J *= 9.0 Hz, H-3′, 5′), 6.84 (1H, s, H-3), 6.77 (1H, s, H-8), 6.37 (1H, s, H-6), 3.87 (3H, s, 7-OMe); ^13^C-NMR (150 MHz, DMSO-*d*_6_): 182.3 (C-4), 165.5 (C-7), 164.5 (C-2), 161.8 (C-4′), 161.6 (C-9), 157.6 (C-5), 133.5 (C-3), 128.9 (C-2′, 6′), 121.4 (C-1′), 116.4 (C-3′, 5′), 103.9 (C-10), 98.7 (C-6), 93.5 (C-8), 56.5 (7-OMe).

Compound** 4**: C_16_H_12_O_6_; ESI/LTQ-Orbitrap-MS* m/z*: 301.07 [M+H]^+^; ^1^H-NMR (600 MHz, DMSO-*d*_6_): 13.04 (5-OH), 7.88 (2H, d,* J *= 8.4 Hz, H-2′, 6′), 6.90 (2H, d,* J *= 8.4 Hz, H-3′, 5′), 6.72 (1H, s, H-3), 6.55 (1H, s, H-8), 3.73 (3H, s, 6-OMe); ^13^C-NMR (150 MHz, DMSO-*d*_6_): 182.3 (C-4), 164.0 (C-2), 161.4 (C-4′), 157.8 (C-7), 153.1 (C-5), 152.7 (C-9), 131.7 (C-6), 128.7 (C-2′, 6′), 121.5 (C-1′), 116.2 (C-3′, 5′), 104.3 (C-3), 102.7 (C-10), 94.6 (C-8), 60.2 (6-OMe).

Compound** 5**: C_17_H_14_O_7_; ESI/LTQ-Orbitrap-MS* m/z*: 331.08 [M+H]^+^; ^1^H-NMR (600 MHz, DMSO-*d*_6_): 13.06 (5-OH), 7.53 (2H, s, H-5′, 6′), 6.91 (1H, d,* J *= 9.0 Hz, H-2′), 6.86 (1H, s, H-3), 6.58 (1H, s, H-8), 3.88 (3H, s, 6-OMe), 3.74 (3H, s, 3′-OMe); ^13^C-NMR (150 MHz, DMSO-*d*_6_): 182.5 (C-4), 164.1 (C-2), 158.1 (C-7), 153.1 (C-9), 152.9 (C-5), 151.2 (C-4′), 148.4 (C-3′), 131.9 (C-6), 121.9 (C-1′), 120.7 (C-6′), 116.2 (C-5′), 110.6 (C-2′), 104.3 (C-10), 103.1 (C-3), 94.8 (C-8), 60.3 (6-OMe), 56.4 (3′-OMe).

Compound** 6**: C_18_H_16_O_8_; ESI/LTQ-Orbitrap-MS* m/z*: 361.09 [M+H]^+^; ^1^H-NMR (600 MHz, DMSO-*d*_6_): 13.04 (5-OH), 7.29 (2H, s, H-2′, 6′), 6.93 (1H, s, H-3), 6.64 (1H, s, H-8), 3.86 (3H, s, 6-OMe), 3.73 (6H, s, 3′, 5′-OMe); ^13^C-NMR (150 MHz, DMSO-*d*_6_): 182.5 (C-4), 164.0 (C-2), 158.1 (C-7), 153.1 (C-5), 152.9 (C-9), 148.6 (C-3′, 5′), 140.3 (C-4′), 131.9 (C-6), 120.9 (C-1′), 104.8 (C-2′, 6′), 104.4 (C-10), 103.5 (C-3), 94.9 (C-8), 60.3 (6-OMe), 56.8 (3′, 5′-OMe).

Compound** 7**: C_18_H_18_O_8_; ESI/LTQ-Orbitrap-MS* m/z*: 375.11 [M+H]^+^; ^1^H-NMR (600 MHz, DMSO-*d*_6_): ^1^H-NMR (600 MHz, DMSO-*d*_6_): 12.91 (5-OH), 7.28 (2H, s, H-2′, 6′), 6.99 (1H, s, H-3), 6.63 (1H, s, H-8), 3.87 (6H, s, 6, 4′-OMe), 3.73 (6H, s, 3′, 5′-OMe); ^13^C-NMR (150 MHz, DMSO-*d*_6_): 182.5 (C-4), 163.4 (C-2), 158.7 (C-7), 153.7 (C-2′, 5′), 153.1 (C-5), 152.5 (C-9), 141.1 (C-4′), 132.1 (C-6), 126.5 (C-1′), 104.9 (C-2′), 104.5 (C-6′), 104.2 (C-10), 103.8 (C-3), 95.2 (C-8), 60.6 (6-OMe), 60.3 (4′-OMe), 56.7 (3′, 5′-OMe).

Compound** 8**: C_22_H_22_O_13_; FAB-MS* m/z*: 495 [M+H]^+^; ^1^H-NMR (600 MHz, DMSO-*d*_6_): 13.01 (5-OH), 7.69 (1H, d,* J *= 2.4 Hz, H-2′), 7.58 (1H, dd,* J *= 2.4, 8.4 Hz, H-6′), 6.86 (1H, d,* J *= 8.4 Hz, H-5′), 6.48 (1H, s, H-8), 5.26 (1H, d,* J *= 7.8 Hz, Glc H-1), 3.86 (3H, s, 6-OMe); ^13^C-NMR (150 MHz, DMSO-*d*_6_): 179.4 (C-4), 158.8 (C-9), 158.5 (C-2), 153.5 (C-5), 153.3 (C-7), 149.5 (C-4′), 145.6 (C-5′), 135.0 (C-3), 132.3 (C-6), 122.9 (C-1′), 122.8 (C-6′), 117.2 (C-2′), 115.7 (C-3′), 105.8 (C-10), 103.9 (Glc C-1), 94.6 (C-8), 78.1 (Glc C-5), 77.8 (Glc C-3), 75.4 (Glc C-2), 70.9 (Glc C-4), 62.2 (Glc C-6), 60.6 (6-OMe).

Compound** 9**: C_22_H_20_O_12_; FAB-MS* m/z*: 465 [M+H]^+^; ^1^H-NMR (600 MHz, DMSO-*d*_6_): 13.01 (5-OH), 7.55 (1H, d,* J *= 1.2 Hz, H-2′), 7.54 (1H, dd,* J *= 1.2, 9.0 Hz, H-6′), 6.84 (1H, d,* J *= 9.0 Hz, H-5′), 6.36 (1H, s, H-8), 6.15 (1H, s, H-6), 5.41 (1H, d,* J *= 7.8 Hz, Glc H-1); ^13^C-NMR (150 MHz, DMSO-*d*_6_): 177.4 (C-4), 161.1 (C-7), 157.2 (C-5), 156.7 (C-2), 156.3 (C-9), 148.6 (C-4′), 144.9 (C-3′), 133.5 (C-3), 121.9 (C-6′), 121.4 (C-1′), 116.3 (C-5′), 115.4 (C-2′), 103.7 (C-10), 101.2 (Glc C-1), 99.2 (C-6), 94.6 (C-8), 77.6 (Glc C-5), 76.5 (Glc C-3), 74.2 (Glc C-2), 70.1 (Glc C-4), 61.0 (Glc C-6).

Compound** 10**: C_27_H_30_O_17_; ESI/LTQ-Orbitrap-MS* m/z*: 649.45 [M+Na]^+^; ^1^H-NMR (600 MHz, DMSO-*d*_6_): ^1^H-NMR (600 MHz, DMSO-*d*_6_): 13.04 (5-OH), 7.64 (1H, d,* J *= 2.4 Hz, H-2′), 7.51 (1H, dd,* J *= 2.4, 8.4 Hz, H-6′), 6.82 (1H, d,* J *= 8.4 Hz, H-5′), 6.36 (1H, s, H-8), 6.14 (1H, s, H-6), 5.31 (1H, d,* J *= 7.8 Hz, Glc H-1), 5.29 (1H, d,* J *= 7.8 Hz, Glc H-1′); ^13^C-NMR (150 MHz, DMSO-*d*_6_): 177.4 (C-4), 165.3 (C-5), 161.2 (C-7), 156.7 (C-2), 155.8 (C-9), 148.6 (C-3′), 144.9 (C-4′), 133.3 (C-3), 122.3 (C-6′), 121.5 (C-1′), 115.9 (C-5′), 115.4 (C-2′), 108.9 (C-10), 103.8 (Glc C-1), 99.2 (Glc C-1′), 98.8 (C-6), 94.0 (C-8), 79.3 (Glc C-5), 77.6 (Glc C-3), 77.5 (Glc C-5′), 77.1 (Glc C-3′), 76.4 (Glc C-2, 2′), 74.2 (Glc C-4), 70.3 (Glc C-4′), 64.3 (Glc C-6), 60.9 (Glc C-6′).

Compound** 11**: C_27_H_30_O_16_; FAB-MS* m/z*: 611 [M+H]^+^; ^1^H-NMR (600 MHz, DMSO-*d*_6_): 13.01 (5-OH), 7.53 (1H, d,* J *= 2.4 Hz, H-2′), 7.51 (1H, dd,* J *= 2.4, 8.4 Hz, H-6′), 6.83 (1H, d,* J *= 8.4 Hz, H-5′), 6.35 (1H, s, H-8), 6.15 (1H, s, H-6), 5.29 (1H, d,* J *= 4.2 Hz, Glc H-1), 4.37 (1H, s, Rham H-1), 0.96 (3H, d,* J *= 6.5 Hz, Rham H-6); ^13^C-NMR (150 MHz, DMSO-*d*_6_): 177.1 (C-4), 164.9 (C-7), 160.8 (C-5), 156.5 (C-2, 9), 148.2 (C-4′), 144.5 (C-3′), 133.2 (C-3), 121.6 (C-6′), 121.1 (C-1′), 116.0 (C-5′), 115.1 (C-2′), 103.5 (C-10), 101.2 (Glc C-1), 100.6 (Rham C-1), 98.9 (C-6), 93.8 (C-8), 76.2 (Glc C-3), 75.7 (Glc C-5), 73.9 (Glc C-2), 71.7 (Rham C-4), 70.4 (Rham C-2), 70.2 (Rham C-3), 69.8 (Glc C-4), 68.2 (Rham C-5), 67.0 (Glc C-6), 17.6 (Rham C-6).

Compound** 12**: C_16_H_18_O_9_; ESI/LTQ-Orbitrap-MS* m/z*: 376.26 [M+Na]^+^; ^1^H-NMR (600 MHz, CD_3_OD): 7.57 (1H, d,* J *= 15.6 Hz, H-7′), 6.97 (1H, d,* J *= 1.8 Hz, H-2′), 6.86 (1H, dd,* J *= 1.8, 7.8 Hz, H-6′), 6.71 (1H, d,* J *= 7.8 Hz, H-5′), 6.25 (1H, d,* J *= 15.6 Hz, H-8′), 5.06 (1H, m, H-3), 4.16 (1H, m, H-5), 3.82 (1H, m, H-4), 2.03–1.79 (4H, m, H-2, 6); ^13^C-NMR (150 MHz, CD_3_OD): 178.2 (1-COOH), 170.5 (C-9′), 151.4 (C-4′), 149.3 (C-7′), 148.9 (C-3′), 129.6 (C-1′), 125.0 (C-6′), 118.4 (C-5′), 117.1 (C-2′), 116.8 (C-8′), 76.5 (C-1), 74.0 (C-3), 72.6 (C-4), 71.2 (C-5), 39.6 (C-2), 39.2 (C-6).

Compound** 13**: C_25_H_24_O_12_; FAB-MS* m/z*: 517 [M+H]^+^; ^1^H-NMR (600 MHz, CD_3_OD): 7.63 (1H, d,* J *= 16.2 Hz, H-7′′), 7.56 (1H, d,* J *= 16.2 Hz, H-7′), 7.08 (1H, d,* J *= 4.2 Hz, H-1′′), 7.06 (1H, d,* J *= 4.2 Hz, H-1′), 7.03 (1H, d,* J *= 1.8 Hz, H-2′), 7.00 (1H, d,* J *= 1.8 Hz, H-2′′), 6.97 (1H, dd,* J *= 1.8, 6.6 Hz, H-6′′), 6.96 (1H, d,* J *= 6.6 Hz, H-5′′), 6.80 (1H, dd,* J *= 1.8, 6.6 Hz, H-6′), 6.78 (1H, d,* J *= 6.6 Hz, H-5′), 6.34 (1H, d,* 2 *= 16.2 Hz, H-8′′), 6.26 (1H, d,* J *= 16.2 Hz, H-8′), 5.44 (1H, m, H-3), 4.31 (1H, d,* J *= 4.2 Hz, H-5), 3.99 (1H, dd,* J *= 3.7, 7.2 Hz, H-4), 2.33 (2H, m, H-6), 2.15 (2H, m, H-2); ^13^C-NMR (150 MHz, CD_3_OD): 177.5 (1-COOH), 169.0 (C-9′′), 168.9 (C-9′), 149.6 (C-4′′), 149.5 (C-4′), 147.8 (C-3′′), 147.4 (C-7′, 7′′), 146.2 (C-3′), 128.0 (C-1′′), 127.9 (C-1′), 123.2 (C-6′′), 123.1 (C-6′), 116.6 (C-5′, 5′′), 115.7 (C-8′′), 115.4 (C-8′), 115.3 (C-2′′), 115.2 (C-2′), 81.0 (C-1), 74.8 (C-4), 72.7 (C-3), 72.2 (C-5), 37.7 (C-6), 36.4 (C-2).

Compound** 14**: C_25_H_24_O_12_; Q-TOF-MS* m/z*: 515.1185 [M−H]^−^; ^1^H-NMR (600 MHz, CD_3_OD): 7.60 (1H, d,* J *= 15.6 Hz, H-7′), 7.52 (1H, d,* J *= 15.6 Hz, H-7′′), 7.03 (1H, d,* J *= 1.8 Hz, H-2′), 7.00 (1H, d,* J *= 1.8 Hz, H-2′′), 6.92 (2H, m, H-6′, 6′′), 6.76 (1H, d,* J *= 7.8 Hz, H-5′), 6.74 (1H, d,* J *= 7.8 Hz, H-5′′), 6.29 (1H, d,* J *= 15.6 Hz, H-8′), 6.20 (1H, d,* J *= 15.6 Hz, H-8′′), 5.65 (1H, m, H-3), 5.12 (1H, dd,* J *= 3.0, 9.0 Hz, H-4), 4.36 (1H, d,* J *= 3.0 Hz, H-5), 2.35 (2H, m, H-6), 2.13 (2H, m, H-2); ^13^C-NMR (150 MHz, CD_3_OD): 177.2 (1-COOH), 170.6 (C-9′), 170.3 (C-9′′), 151.5 (C-4′, 4′′), 149.7 (C-7′), 149.5 (C-7′′), 148.6 (C-3′, 3′′), 129.6 (C-1′), 129.5 (C-1′′), 125.1 (C-6′, 6′′), 118.4 (C-5′, 5′′), 117.1 (C-2′, 2′′), 116.6 (C-8′, 8′′), 78.0 (C-1), 77.9 (C-4), 71.5 (C-5), 71.0 (C-3), 41.4 (C-2), 40.3 (C-6).

Compound** 15**: C_25_H_24_O_12_; Q-TOF-MS* m/z*: 515.1185 [M−H]^−^; ^1^H-NMR (600 MHz, CD_3_OD): 7.62 (1H, d,* J *= 16.2 Hz, H-7′), 7.58 (1H, d,* J *= 16.2 Hz, H-7′′), 7.07 (2H, t,* J *= 1.8, 4.2 Hz, H-2′, 2′′), 6.96 (2H, m, H-6′, 6′′), 6.75 (1H, d,* J *= 7.8 Hz, H-5′), 6.72 (1H, d,* J *= 7.8 Hz, H-5′′), 6.36 (1H, d,* J *= 16.2 Hz, H-8′), 6.28 (1H, d,* J *= 16.2 Hz, H-8′′), 5.41 (1H, m, H-3), 5.39 (1H, m, H-5), 3.98 (1H, dd,* J *= 3.6, 7.8 Hz, H-4), 2.35 (2H, m, H-6), 2.20 (2H, m, H-2); ^13^C-NMR (150 MHz, CD_3_OD): 179.4 (1-COOH), 170.9 (C-9′), 170.4 (C-9′′), 151.5 (C-4′), 151.4 (C-4′′), 149.2 (C-7′, 7′′), 149.0 (C-3′), 148.6 (C-3′′), 129.8 (C-1′), 129.7 (C-1′′), 125.0 (C-6′), 124.9 (C-6′′), 118.4 (C-5′, 5′′), 117.5 (C-8′), 117.2 (C-8′′), 117.1 (C-2′), 117.0 (C-2′′), 76.7 (C-1), 74.5 (C-5), 74.0 (C-3), 72.6 (C-4), 39.7 (C-2), 38.0 (C-6).

Compound** 16**: C_26_H_26_O_12_; Q-TOF-MS* m/z*: 529.1349 [M−H]^−^; ^1^H-NMR (600 MHz, CD_3_OD): 7.63 (1H, d,* J *= 16.2 Hz, H-7′), 7.56 (1H, d,* J *= 16.2 Hz, H-7′′), 7.07 (1H, d,* J *= 2.4 Hz, H-2′), 7.06 (1H, d,* J *= 2.4 Hz, H-2′′), 6.97 (2H, m, H-6′, 6′′), 6.80 (1H, d,* J *= 8.4 Hz, H-5′), 6.78 (1H, d,* J *= 8.4 Hz, H-5′′), 6.35 (1H, d,* J *= 16.2 Hz, H-8′), 6.23 (1H, d,* J *= 16.2 Hz, H-8′′), 5.41 (1H, m, H-3), 5.32 (1H, m, H-5), 3.98 (1H, dd,* J *= 3.0, 6.6 Hz, H-4), 3.69 (3H, s, 1-COOMe), 2.35 (2H, m, H-6), 2.20 (2H, m, H-2); ^13^C-NMR (150 MHz, CD_3_OD): 177.5 (1-COOMe), 170.6 (C-9′), 169.8 (C-9′′), 151.6 (C-4′), 151.4 (C-4′′), 149.3 (C-7′), 149.0 (C-7′′), 148.7 (C-3′), 148.6 (C-3′′), 129.7 (C-1′), 129.4 (C-1′′), 124.9 (C-6′), 124.8 (C-6′′), 118.4 (C-5′), 118.3 (C-5′′), 117.2 (C-8′, 8′), 117.0 (C-2′, 2′) 76.5 (C-1), 74.0 (C-5), 73.8 (C-3), 72.6 (C-4), 54.9 (1-COOMe), 38.5 (C-2), 37.5 (C-6).

Compound** 17**: C_26_H_44_O_8_; ESI/LTQ-Orbitrap-MS* m/z*: 507.29 [M+Na]^+^; ^1^H-NMR (600 MHz, Pyridine-*d*_6_): 5.06 (1H, d,* J *= 8.4 Hz, Glc H-1), 4.52 (1H, d,* J *= 10.6 Hz, H-17a), 4.12 (1H, t, H-3), 3.95 (1H, d,* J *= 10.6 Hz, H-17b), 2.43 (1H, s, H-14a), 2.02 (1H, m, H-13), 1.94 (1H, m, H-2a), 1.88 (1H, m, H-1a), 1.82 (1H, m, H-15a), 1.78 (1H, m, H-14b), 1.66 (1H, m, H-7a), 1.65 (2H, m, H-11), 1.64 (1H, m, H-6a), 1.57 (1H, m, H-5), 1.47 (1H, m, H-7b), 1.42 (1H, m, H-6b), 1.35 (1H, m, 15b), 1.34 (1H, m, H-2b), 1.21 (3H, s, 20), 1.02 (3H, s, 19), 0.99 (3H, s, 18), 0.98 (1H, m, H-9), 0.89 (1H, m, 1b); ^13^C-NMR (150 MHz, pyridine-*d*_6_): 105.7 (Glc C-1), 82.5 (C-16), 80.1 (C-3), 78.4 (Glc C-5), 78.3 (Glc C-3), 75.7 (Glc C-2), 75.5 (C-17), 72.1 (Glc C-4), 63.2 (Glc C-6), 58.6 (C-9), 56.9 (C-5), 54.0 (C-15), 47.2 (C-13), 46.0 (C-8), 43.6 (C-7), 40.6 (C-10), 40.4 (C-4), 40.3 (C-1), 38.5 (C-14), 29.3 (C-19), 28.4 (C-2), 27.7 (C-12), 21.7 (C-6), 19.9 (C-11), 18.8 (C-20), 16.6 (C-18).

Compound** 18**: C_26_H_44_O_8_; ESI/LTQ-Orbitrap-MS* m/z*: 507.29 [M+Na]^+^; ^1^H-NMR (600 MHz, Pyridine-*d*_6_): 5.05 (1H, d,* J *= 7.8 Hz, Glc H-1), 4.49 (1H, d,* J *= 10.6 Hz, H-17a), 4.12 (1H, t, H-3), 3.92 (1H, d,* J *= 10.6 Hz, H-17b), 2.45 (1H, s, H-14a), 2.02 (1H, m, H-13), 1.94 (1H, m, H-2a), 1.88 (1H, m, H-1a), 1.82 (1H, m, H-15a), 1.78 (1H, m, H-14b), 1.66 (1H, m, H-7a), 1.65 (2H, m, H-11), 1.64 (1H, m, H-6a), 1.57 (1H, m, H-5), 1.47 (1H, m, H-7b), 1.42 (1H, m, H-6b), 1.35 (1H, m, 15b), 1.34 (1H, m, H-2b), 1.18 (3H, s, 20), 1.02 (3H, s, 19), 0.98 (1H, m, H-9), 0.89 (1H, m, 1b), 0.88 (3H, s, 18); ^13^C-NMR (150 MHz, Pyridine-*d*_6_): 107.4 (Glc C-1), 81.5 (C-16), 79.4 (Glc C-5), 79.3 (Glc C-3), 76.4 (Glc C-2), 76.2 (C-17), 75.8 (C-3), 72.3 (Glc C-4), 63.5 (Glc C-6), 57.5 (C-9), 54.0 (C-15), 49.6 (C-5), 47.2 (C-13), 45.5 (C-8), 43.1 (C-7), 40.2 (C-10), 38.7 (C-4), 38.2 (C-14), 34.5 (C-1), 30.1 (C-18), 27.6 (C-2), 27.1 (C-12), 23.1 (C-19), 21.1 (C-6), 19.2 (C-20), 18.6 (C-11).

Compound** 19**: C_26_H_42_O_8_; FAB-MS* m/z*: 481 [M−H]^−^; ^1^H-NMR (600 MHz, DMSO-*d*_6_): 4.16 (1H, d,* J *= 7.8 Hz, Glc H-1), 4.03 (1H, d,* J *= 10.8 Hz, H-17a), 3.38 (1H, d,* J *= 10.8 Hz, H-17a), 2.43 (1H, m, H-2a), 1.99 (1H, m, H-13), 1.92 (1H, m, H-1), 1.82 (1H, m, H-2b), 1.77 (2H, m, H-14), 1.58 (1H, m, H-5), 1. 57 (1H, m, H-15a), 1.56 (2H, m, H-11), 1.51 (2H, m, H-12), 1.45 (2H, m, H-7), 1.40 (2H, m, H-6), 1.35 (1H, m, H-15b), 1.07 (1H, m, H-9), 0.99 (6H, s, H-18, 20), 0.93 (3H, s, H-19); ^13^C-NMR (150 MHz, DMSO-*d*_6_): 218.7 (C-3), 105.8 (Glc C-1), 81.2 (C-16), 81.0 (Glc C-5), 77.6 (Glc C-3), 75.3 (Glc C-2), 75.1 (C-17), 71.5 (Glc C-4), 62.5 (Glc C-6), 56.3 (C-9), 54.8 (C-5), 53.2 (C-15), 48.0 (C-4), 46.3 (C-13), 45.3 (C-8), 42.1 (C-7), 40.1 (C-1), 39.5 (C-10), 37.8 (C-14), 35.1 (C-2), 28.4 (C-18), 27.3 (C-12), 22.8 (C-6), 22.2 (C-19), 19.9 (C-11), 19.0 (C-20).

Compound** 20**: C_15_H_20_O_9_; FAB-MS* m/z*: 335 [M+H]^+^; ^1^H-NMR (600 MHz, CD_3_OD): 6.27 (1H, d,* J *= 2.4 Hz, H-5), 6.19 (1H, d,* J *= 2.4 Hz, H-3), 5.04 (1H, d,* J *= 7.8 Hz, Glc H-1), 3.89 (3H, s, 6-OMe), 2.59 (3H, s, COCH_3_); ^13^C-NMR (150 MHz, CD_3_OD): 206.2 (COCH_3_), 166.7 (C-2), 165.2 (C-4), 164.8 (C-6), 107.9 (C-1), 100.8 (Glc C-1), 97.4 (C-3), 93.2 (C-5), 77.9 (Glc C-3), 77.2 (Glc C-5), 74.3 (Glc C-2), 70.9 (Glc C-4), 62.0 (Glc C-6), 56.7 (6-OMe), 33.3 (COCH_3_).

### 3.2. AR Inhibitory Activities of the Extracts, Fractions, and Compounds 1–20 from* A. iwayomogi*

Our study investigated the inhibitory effects of* A. iwayomogi* on AR and AGEs formation, which demonstrates the potential of these compounds to prevent diabetes complications. The extracts and fractions of the aerial parts of the* A. iwayomogi *were tested for AR inhibition. The results are summarized in Table 1. The extracts, 30%, 60%, and 100% MeOH fractions from* A. iwayomogi* showed significant inhibition of AR (IC_50_ value with 2.71 ± 0.72, 1.16 ± 0.10, 1.42 ± 0.17, and 0.79 ± 0.15 *μ*g/mL, resp.). In addition, compounds** 1**–**20 **were tested for their ability to inhibit AR. As can be seen in Table 2, genkwanin (**3**), the three diterpene glycosides (**17**–**19**), and 2,4-dihydroxy 6-methoxyacetophenone 4-*O*-*β*-d-glucopyranoside (**20**) demonstrated no AR inhibition; all had IC_50_ values >200 *μ*M. On the other hand, hispidulin (**4**), patuletin-3-*O*-glucoside (**8**), isoquercetin (**9**), and five caffeoylquinic acids (**12**,** 13**,** 14**,** 15**, and** 16**) were stronger inhibitors than TMG (IC_50_ value with 6.58 ± 0.19 *μ*M), which was used as the positive control, as a compound known to inhibit AR. The IC_50_ values of compounds** 4**,** 8**,** 9**,** 12**,** 13**,** 14**,** 15,** and** 16** were 2.93 ± 0.11, 3.19 ± 0.10, 3.32 ± 0.21, 4.54 ± 0.16, 0.22 ± 0.01, 1.90 ± 0.01, 1.25 ± 0.01, and 1.48 ± 0.04 *μ*M, respectively.

### 3.3. Inhibitory Activities of the Extracts, Fractions, and Compounds 1–20 from* A. iwayomogi* on AGEs Formation

The extracts and fractions from* A. iwayomogi* were investigated for inhibition of AGEs formation. The results are shown in Table 1. The extracts, CHCl_3_, 30% MeOH, and 100% MeOH fractions all showed greater inhibition than AG (IC_50_ value with 45.85 ± 7.17 *μ*g/mL), the positive control. The IC_50_ values of extracts, CHCl_3_, 30% MeOH, and 100% MeOH fractions were 40.99 ± 0.50, 7.29 ± 0.05, 40.81 ± 0.05, and 38.40 ± 0.51 *μ*g/mL, respectively. Compounds** 1**–**20** isolated from* A. iwayomogi* were also tested for inhibition of AGEs formation, as reported in Table 2. Similar to AR inhibitory activities, diterpene glycosides (**17**–**19**) showed no inhibition of AGEs formation and compound** 20** showed only slight inhibition, whereas the two coumarins (**1** and** 2**), nine flavonoids (**3**–**11**), and five caffeoylquinic acids (**12**–**16**) were all more inhibitory than AG (IC_50_ value with 414.77 ± 5.96 *μ*M). Among them, compound** 1**, which exhibited minor inhibition on AR, exhibited the best inhibition of AGEs formation with an IC_50_ value of 20.71 ± 1.38 *μ*M.

### 3.4. Simultaneous Quantitative Analysis of Bioactive Compounds in* A. iwayomogi* to Select Suitable Region in Korea

**Figure 2:**
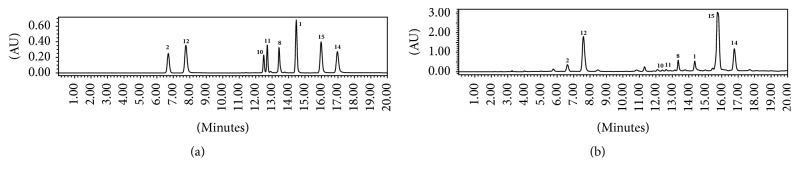
Chromatograms of standards mixture (a) and* A. iwayomogi* ext. (b).

**Figure 3:**
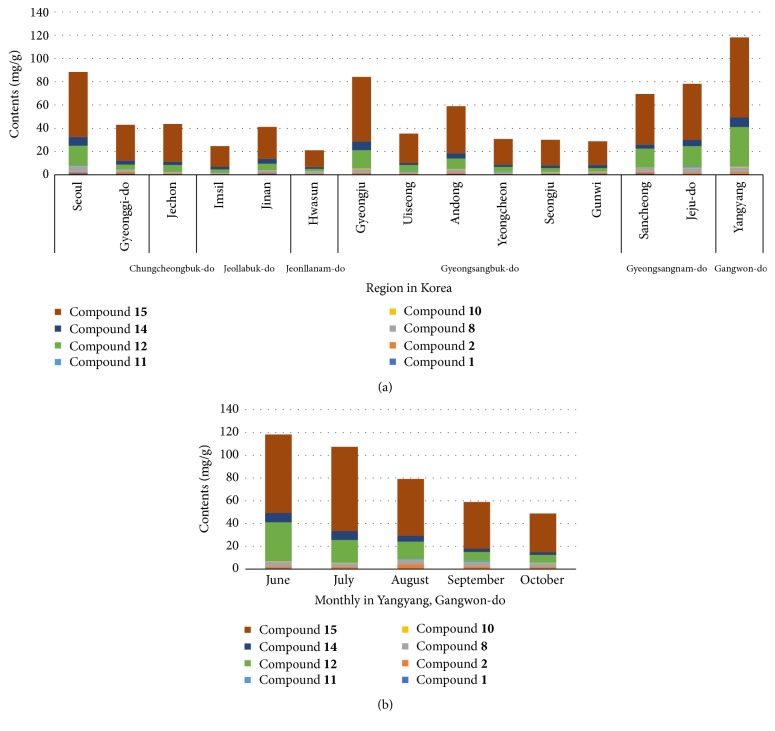
Total contents of compounds** 1**,** 2**,** 8**,** 10**,** 11**,** 12**,** 14, **and** 15 **in* A. iwayomogi* by region (a) and by month in Yangyang, Gangwon-do, Korea (b).

Since* A. iwayomogi* is found mainly in Korea, we collected 15 samples of* A. iwayomogi* in different regions including Seoul, Gyeongii-do, Chungcheongbuk-do, Jeollabuk-do, Jeollanam-do, Gyeongsangbuk-do, Gyeongsangnam-do, Gangwon-do, and Jeju-do. The following compounds were analyzed by HPLC-PAD from these fifteen specimens: scopoletin (**1**), scopolin (**2**), patulein-3-*O*-glucoside (**8**), quercetin-3-gentiobioside (**10**), rutin (**11**), 3-*O*-caffeoylquinic acid (**12**), 3,4-di-*O*-caffeoylquinic acid (**14**), and 3,5-di-*O*-caffeoylquinic acid (**15**). They all showed significant inhibition of either AR or AGEs formation. Compounds** 1**,** 2**,** 8**,** 10**,** 11**,** 12**,** 14**, and** 15 **were detected in 14.4, 6.6, 13.3, 12.4, 12.7, 7.6, 16.9, and 15.9 min, respectively (Figure 2). To optimize analytical methods, the standard calibration curve, limit of detection (LOD), and limit of quantification (LOQ) for compounds** 1**,** 2**,** 8**,** 10**,** 11**,** 12**,** 14**, and** 15** are measured. The standard calibration curves of compounds** 1**,** 2**,** 8**,** 10**,** 11**,** 12**,** 14**, and** 15 **were detected at linear range 0.1–1000 *μ*g/mL. As a result, the correlation coefficient (*r*^2^) of all bioactive compounds is above 0.999. The values of LOD of compounds** 1**,** 2**,** 8**,** 10**,** 11**,** 12**,** 14**, and** 15 **are shown to be 2.80, 0.50, 5.93, 5.08, 4.99, 3.82, 1.81, and 2.38 and the values of LOQ of bioactive compounds are shown to be 8.49, 1.52, 17.98, 15.41, 15.12, 11.59, 5.48, and 7.21 *μ*g/mL, respectively. Using optimized analytical methods, the amounts of bioactive compounds from* A. iwayomogi* in the 15 samples were determined (Table 3). As a result, contents of compounds** 1** and** 8** were the highest in Seoul (1.38 ± 0.01, 4.80 ± 0.03 mg/g, resp.), compound** 10** was the highest in Gyeongju, Gyeongsangbuk-do (0.65 ± 0.06 mg/g), and compound** 11** was highest in Jeju-do (1.11 ± 0.07 mg/g). Furthermore, compounds** 2**,** 12**,** 14**, and** 15** were the most abundant in Yangyang, Gangwon-do (1.67 ± 0.02, 33.61 ± 0.48, 8.18 ± 0.10, and 68.87 ± 0.19 mg/g, resp.). Compound** 2** exhibited good inhibition activity of AGEs formation while compounds** 12**,** 14**, and** 15 **were significant inhibitors of both AR and AGEs formation. Overall, the specimen with the highest total contents of the most compounds was acquired in Yangyang, Gangwon-do (Figure 3). We therefore collected new specimens of* A. iwayomogi* in June, July, August, September, and October in Yangyang, Gangwon-do, and then analyzed the amounts of bioactive compounds in each specimen using HPLC (Table 4). Amounts of compounds** 1**,** 10**,** 12**, and** 14** were taken in June (0.74 ± 0.02, 0.59 ± 0.12, 33.61 ± 0.48, and 8.18 ± 0.10 mg/g, resp.), that of compound** 15** was taken in July (73.99 ± 0.35 mg/g), those of compounds** 2** and** 8** were taken in August (3.67 ± 0.02 and 4.16 ± 0.04 mg/g, resp.), and that of compound** 11** was taken in September (0.86 ± 0.02 mg/g).

## 4. Discussion

The worldwide prevalence of diabetes has become a massive health burden significantly decreasing quality of life and increasing morbidity, all at a huge economic cost. As diabetes is recognized as a serious disease, diabetic complications are also recognized as a serious disease. Accordingly, preventing treatment for diabetic complications is as important as diabetes and blocking the polyol pathway plays an important role preventing diabetic complications. Therefore, AR and AGEs formation inhibitors significantly affected diabetic complications.* Artemisia iwayomogi*, locally called haninjin or dowijigi, is a member of the Compositae family and a perennial herb mainly found in Korea.* A. iwayomogi* has long been used in Korea in foods such as tea, rice cake, and soup and for the treatment of various diseases. However, previous literatures did not have reported on inhibitory activities of* A. iwayomogi* on AR and AGEs formation. For these reasons, we aimed to assess* Artemisia iwayomogi*, which is native plant in Korea used as usual food, and has potential as anti-diabetic complications agent to inhibit AR and AGEs formation.

Our research led to the isolation of two coumarins (**1** and** 2**), nine flavonoids (**3**–**11**), five caffeoylquinic acids (**12**–**16**), three diterpene glycosides (**17**–**19**), and one phenolic compound (**20**). Among them, compounds** 4**,** 6**,** 7**,** 10**,** 13**, and** 18** were first reported on the isolation from* A. iwayomogi*. Compounds** 1**–**20** have various bioactive properties such as antioxidant, anti-inflammation, antiangiogenesis, and cardiovascular activity [35–37].

After that, the extracts, fractions, and compounds** 1**–**20** from* A. iwayomogi* were investigated for inhibition of AR. The extracts, 30%, 60%, and 100% MeOH fractions from* A. iwayomogi* showed significant inhibition of AR more than TMG, positive control. Among compounds isolated from* A. iwayomogi*, compounds** 4**,** 8**,** 9**,** 12**,** 13**,** 14**,** 15**, and** 16** were stronger inhibitors than TMG. Out of these, compound** 13** exhibited the strongest inhibition. Previous literature reported that caffeic acid had no inhibitory activity on AR but that caffeoyl derivatives are significant inhibitors of AR [24, 38]. Existence of caffeoyl groups for substituents such as quinicacid is important parameter for not only AR inhibitory activity but also various biological activities [39]. Furthermore, other studies have demonstrated relationships between the inhibitory activity and structure of flavonoids [4]. They reported that monohydroxyflavones (such as compound** 3**) are not inhibitors of AR, that flavonoid glucosides (compounds** 8** and** 9**) are more active than flavonoid diglucosides (compounds** 10** and** 11**), and that flavonoids with a substituted hydroxyl group (compound** 6**) are more active than those with substituted methoxyl groups (compound** 7**). Inhibition of AR by flavonoids isolated from* A. iwayomogi* in our study demonstrated similar results as previous studies. We demonstrated the inhibitory activities of not only AR but also AGEs formation. The CHCl_3_ fraction from* A. iwayomogi* had no inhibitory activity on AR but showed the best inhibition on AGEs formation. The two coumarins (**1** and** 2**), nine flavonoids (**3**–**11**), and five caffeoylquinic acids (**12**–**16**) exhibited also great inhibitory activities more than AG, positive control. Among them, compound** 1** showed minor inhibition on AR and exhibited the best inhibition of AGEs formation. Previous literature has reported that the presence of a methoxyl group at C-6 instead of a hydroxyl group and a hydroxyl group at C-7 in the coumarin structure are important parameters in the inhibition of AGEs formation [40]. Therefore, compound** 1** may be more active than coumarins with other substituents.

Since* A. iwayomogi* is found mainly in Korea, we collected 15 samples of* A. iwayomogi* in different regions. The following compounds were analyzed by HPLC-PAD from these fifteen specimens: scopoletin (**1**), scopolin (**2**), patulein-3-*O*-glucoside (**8**), quercetin-3-gentiobioside (**10**), rutin (**11**), 3-*O*-caffeoylquinic acid (**12**), 3,4-di-*O*-caffeoylquinic acid (**14**), and 3,5-di-*O*-caffeoylquinic acid (**15**). They all showed significant inhibition of either AR or AGEs formation. Overall, the most abundant bioactive compounds were harvested in Yangyang, Gangwon-do, from June (Figure 3). We demonstrated that* A. iwayomogi*, which was harvested in Yangyang, Gangwon-do, from June, was the best used for medicinal food such as diabetic complications.

## 5. Conclusion

In summary, our study reported the isolation of two coumarins (**1** and** 2**), nine flavonoids (**3**–**11**), five caffeoylquinic acids (**12**–**16**), three diterpene glycosides (**17**–**19**), and one phenolic compound (**20**). Among them, hispidulin (**4**), 6-methoxytricin (**6**), arteanoflavone (**7**), quercetin-3-gentiobioside (**10**), 1,3-di-*O*-caffeoylquinic acid (**13**), and suavioside A (**18**) were isolated for the first time from* A. iwayomogi*. In addition, two coumarins, nine flavonoids, and five caffeoylquinic acids isolated from* A. iwayomogi *demonstrated biological activities against AR and AGEs formation. Since extracts of* A. iwayomogi* contained enough amounts of active compounds,* A. iwayomogi* extract can be used for the successful crude herbal drug against AR and AGEs formation. Because* A. iwayomogi* is native to Korea, we analyzed the bioactive compounds by region. We determined that it is best to harvest* A. iwayomogi* in Yangyang, Gangwon-do, from June in order to get the highest content of active compounds. Based on these, our research demonstrates that* A. iwayomogi *containing active compounds is clearly a potential candidate for a new natural alternative medicine or health supplement and a preventive or therapeutic agent in the treatment of diabetic complications.

## Figures and Tables

**Table 1:** IC_50_ of the extraction and fractions from *A. iwayomogi *on aldose reductase (AR) and advanced glycation endproducts (AGEs) formation.

Sample	IC_50_^*a*^ (*μ*g/ml)	
	AR	AGEs formation
50% EtOH ext.	2.71 ± 0.72^*∗∗*^	40.99 ± 0.50^*∗*^
CHCl_3_ fr.	15.36 ± 3.64^*∗∗*^	7.29 ± 0.05^*∗*^
30% MeOH fr.	1.16 ± 0.10^*∗*^	40.81 ± 0.05^*∗*^
60% MeOH fr.	1.42 ± 0.17^*∗*^	93.76 ± 0.06^*∗*^
100% MeOH fr.	0.79 ± 0.15^*∗*^	38.40 ± 0.51^*∗*^
Water fr.	19.67 ± 0.81^*∗∗*^	402.45 ± 4.28^*∗∗*^
TMG^*b*^	1.22 ± 0.03^*∗*^	—
AG^*c*^	—	45.85 ± 7.17^*∗∗*^

^*a*^IC_50_ calculated from the least-squares regression line of the logarithmic concentrations plotted against the residual activity.

^*c*^TMG was used as a positive control.

^*c*^AG was used as a positive control.

^*∗*^Significant difference from control; ^*∗*^*p* < 0.05, ^*∗∗*^*p* < 0.01.

**Table 2:** IC_50_ of compounds **1**–**20** isolated from *A. iwayomogi *on aldose reductase (AR) and advanced glycation endproducts (AGEs) formation.

Compound	IC_50_^*a*^ (*μ*M)	
	AR	AGEs formation
1	90.39 ± 1.91^*∗∗*^	20.71 ± 1.38^*∗∗*^
**2**	131.37 ± 0.12^*∗*^	82.43 ± 1.52^*∗∗*^
**3**	>200	311.89 ± 11.26^*∗∗*^
**4**	2.93 ± 0.11^*∗∗*^	185.56 ± 0.20^*∗∗*^
**5**	110.65 ± 9.89^*∗∗*^	169.61 ± 16.41^*∗∗*^
**6**	30.29 ± 3.95^*∗*^	134.88 ± 0.84^*∗∗*^
**7**	102.53 ± 7.81^*∗∗*^	207.70 ± 13.32^*∗∗*^
**8**	3.19 ± 0.10^*∗∗*^	197.38 ± 11.03^*∗∗*^
**9**	3.32 ± 0.21^*∗∗*^	67.42 ± 9.95^*∗∗*^
**10**	10.60 ± 0.38^*∗∗*^	109.46 ± 1.41^*∗∗*^
**11**	12.53 ± 0.20^*∗∗*^	129.15 ± 4.01^*∗∗*^
**12**	4.54 ± 0.16^*∗∗*^	176.16 ± 5.77^*∗∗*^
**13**	0.22 ± 0.01^*∗∗*^	24.85 ± 1.86^*∗∗*^
**14**	1.90 ± 0.01^*∗∗*^	124.28 ± 4.25^*∗∗*^
**15**	1.25 ± 0.01^*∗∗*^	31.78 ± 0.56^*∗∗*^
**16**	1.48 ± 0.04^*∗∗*^	44.45 ± 4.86^*∗∗*^
**17**	>200	ND^*d*^
**18**	>200	ND^*d*^
**19**	>200	ND^*d*^
**20**	>200	1048.99 ± 0.11^*∗*^
TMG^*b*^	6.58 ± 0.19^*∗*^	—
AG^*c*^	—	414.77 ± 5.96^*∗*^

^*a*^IC_50_ calculated from the least-squares regression line of the logarithmic concentrations plotted against the residual activity.

^*b*^TMG was used as a positive control.

^*c*^AG was used as a positive control.

^*d*^ND was not detected.

^*∗*^Significant difference from control; ^*∗*^*p* < 0.05, ^*∗∗*^*p* < 0.01.

**Table 3:** Contents of compounds **1**, **2**, **8**,** 10**, **11**, **12**, **14, **and** 15** in *A. iwayomogi* by region in Korea.

Sample	Contents (mg/g)							
	**1**	**2**	**8**	**10**	**11**	**12**	**14**	**15**
Seoul	1.38 ± 0.01	1.16 ± 0.02	4.80 ± 0.03	0.25 ± 0.20	0.69 ± 0.42	16.79 ± 0.19	7.43 ± 0.18	55.86 ± 0.79
Gyeonggi-do	0.98 ± 0.01	1.49 ± 0.01	1.64 ± 0.03	0.23 ± 0.07	0.23 ± 0.01	4.18 ± 0.05	3.40 ± 0.01	30.83 ± 0.06
Jechon, Chungcheongbuk-do	0.57 ± 0.10	0.43 ± 0.01	1.25 ± 0.06	0.22 ± 0.02	0.27 ± 0.01	5.62 ± 0.05	2.90 ± 0.02	32.44 ± 0.28
Imsil, Jeollabuk-do	0.26 ± 0.01	0.30 ± 0.01	0.96 ± 0.04	0.14 ± 0.02	0.38 ± 0.04	2.56 ± 0.22	2.23 ± 0.03	17.82 ± 0.07
Jinan, Jeollabuk-do	0.96 ± 0.04	0.70 ± 0.01	1.85 ± 0.01	0.36 ± 0.02	0.25 ± 0.04	5.46 ± 0.17	4.11 ± 0.03	27.51 ± 0.43
Hwasun, Jeonllanam-do	0.19 ± 0.01	0.62 ± 0.01	1.99 ± 0.09	0.26 ± 0.03	0.32 ± 0.02	1.66 ± 0.14	1.68 ± 0.01	14.33 ± 0.64
Gyeongju, Gyeongsangbuk-do	0.63 ± 0.10	0.75 ± 0.03	3.45 ± 0.11	0.65 ± 0.06	0.34 ± 0.05	15.28 ± 0.15	7.58 ± 0.21	55.49 ± 0.17
Uiseong, Gyeongsangbuk-do	0.50 ± 0.01	0.61 ± 0.03	1.27 ± 0.02	0.07 ± 0.05	0.43 ± 0.26	5.45 ± 0.23	1.63 ± 0.01	25.44 ± 0.05
Andong, Gyeongsangbuk-do	0.94 ± 0.01	0.98 ± 0.01	2.34 ± 0.02	0.41 ± 0.11	0.43 ± 0.01	8.94 ± 0.06	4.49 ± 0.03	40.52 ± 0.13
Yeongcheon, Gyeongsangbuk-do	0.29 ± 0.01	0.43 ± 0.01	1.21 ± 0.03	0.10 ± 0.03	0.51 ± 0.02	4.32 ± 0.15	1.94 ± 0.01	21.99 ± 0.27
Seongju, Gyeongsangbuk-do	0.73 ± 0.03	0.29 ± 0.01	1.18 ± 0.01	0.20 ± 0.02	0.27 ± 0.01	3.07 ± 0.09	2.36 ± 0.03	21.92 ± 0.07
Gunwi, Gyeongsangbuk-do	0.67 ± 0.01	0.75 ± 0.01	1.20 ± 0.01	0.20 ± 0.03	0.18 ± 0.01	2.79 ± 0.07	2.58 ± 0.04	20.46 ± 0.01
Sancheong, Gyeongsangnam-do	1.05 ± 0.01	1.56 ± 0.02	3.25 ± 0.02	0.31 ± 0.06	0.39 ± 0.04	15.94 ± 0.49	3.46 ± 0.08	43.47 ± 0.05
Jeju-do	0.12 ± 0.03	1.59 ± 0.02	4.03 ± 0.10	0.35 ± 0.10	1.11 ± 0.07	17.45 ± 0.19	5.34 ± 0.03	48.22 ± 0.95
Yangyang, Gangwon-do	0.74 ± 0.02	1.67 ± 0.02	3.95 ± 0.45	0.59 ± 0.12	0.53 ± 0.02	33.61 ± 0.48	8.18 ± 0.10	68.87 ± 0.19

Data are mean ± SD (*n* = 3) in mg/g dried sample.

**Table 4:** Contents of compounds **1**, **2**, **8**,** 10**, **11**, **12**, **14, **and** 15** from *A. iwayomogi* in Yangyang, Gangwon-do by month.

Sample	Contents (mg/g)							
	**1**	**2**	**8**	**10**	**11**	**12**	**14**	**15**
June	0.74 ± 0.02	1.67 ± 0.02	3.95 ± 0.45	0.59 ± 0.12	0.53 ± 0.02	33.61 ± 0.48	8.18 ± 0.10	68.87 ± 0.19
July	0.08 ± 0.01	1.81 ± 0.01	3.19 ± 0.08	0.31 ± 0.04	0.47 ± 0.01	19.74 ± 0.08	7.73 ± 0.02	73.99 ± 0.35
August	0.28 ± 0.01	3.67 ± 0.02	4.16 ± 0.04	0.30 ± 0.04	0.67 ± 0.09	15.15 ± 0.01	4.97 ± 0.08	49.99 ± 0.99
September	0.20 ± 0.01	2.24 ± 0.01	3.71 ± 0.01	0.21 ± 0.02	0.86 ± 0.02	7.81 ± 0.13	2.88 ± 0.01	40.97 ± 0.50
October	0.19 ±0.01	1.58 ± 0.01	3.42 ± 0.06	0.30 ± 0.02	0.34 ± 0.01	6.63 ± 3.07	2.25 ± 0.01	34.03 ± 0.09

Data are mean ± SD (*n* = 3) in mg/g dried sample.
